# Altered intrinsic insular activity predicts symptom severity in unmedicated obsessive-compulsive disorder patients: a resting state functional magnetic resonance imaging study

**DOI:** 10.1186/s12888-016-0806-9

**Published:** 2016-04-16

**Authors:** Yajing Zhu, Qing Fan, Haiyin Zhang, Jianyin Qiu, Ling Tan, Zeping Xiao, Shanbao Tong, Jue Chen, Yao Li

**Affiliations:** Shanghai Mental Health Center, Shanghai Jiao Tong University School of Medicine, Shanghai, China; Med-X Research Institute, School of Biomedical Engineering, Shanghai Jiao Tong University, Shanghai, China; Department of Radiology, Ruijin Hospital, Shanghai Jiao Tong University School of Medicine, Shanghai, China

**Keywords:** Obsessive-compulsive disorder, Resting-state functional magnetic resonance imaging, Salience network, Insula, Spontaneous neuronal activity

## Abstract

**Background:**

Previous neuroimaging data indicated that the dysfunction in cortico-striato-thalamo-cortical (CSTC) circuit contributed to the neuropathological mechanism of obsessive-compulsive disorder (OCD). Whereas, emerging work has shown that the pathophysiology of OCD might be related to more widely distributed large-scale brain systems including limbic system and the salience network. This study aims to investigate the aberrant spontaneous neuronal activity within the whole brain, and its association with the symptom severity for unmedicated OCD patients.

**Method:**

Twenty-eight unmedicated OCD adults and twenty-eight matched healthy controls were recruited for a resting state functional magnetic resonance imaging (fMRI) study. The amplitude of low-frequency fluctuation (ALFF) analysis over whole brain was performed to examine the intrinsic cerebral activity of subjects. In addition, we conducted the voxel-based Pearson’s correlative analysis to probe into the relationship between ALFF values and symptom severity for OCD patients.

**Results:**

Our results showed that OCD patients had increased ALFF measures in the left frontopolar cortex and left orbital frontal cortex (OFC), with decreased ALFF values in the right insula. Moreover, the right insular intrinsic activity was significantly correlated with total YBOCS score (*r* = 0.611, *p* = 0.002) and compulsion score (*r* = 0.640, *p* = 0.001) for OCD patients.

**Conclusion:**

The results showed abnormal intrinsic neuronal activity within CSTC circuit and salience network of OCD patients. Our finding of aberrant insular activity advanced the understanding of OCD pathophysiology beyond the traditional CSTC circuit. To the best of our knowledge, it is the first finding about a reduced insular activity at the resting state for unmedicated OCD patients, which might serve as an informative biomarker for OCD pathophysiology.

## Background

Obsessive-compulsive disorder (OCD) is a chronic neuropsychiatric disorder characterized by intrusive thoughts and repetitive behaviors engaged to reduce the associated anxiety [1, 2]. Despite its high prevalence, the pathogenesis of OCD remains not fully understood. A variety of neuroimaging studies reveal that the cortico-striato-thalamo-cortical (CSTC) pathway dysfunction plays an important role in the pathophysiology of OCD [3, 4]. A meta-analysis of magnetic resonance imaging (MRI) data reported the structural alterations in OCD patients, including reduced volume in both anterior cingulate cortex (ACC) and orbital frontal cortex (OFC) areas, and enhanced volume in thalamus [5]. From a battery of functional MRI (fMRI) studies, hyperactivities were observed in the CSTC circuit, especially within prefrontal regions including ACC, OFC, dorsolateral prefrontal cortex (DLPFC), and subcortical areas including caudate and thalamus [6, 7].

However, emerging evidence has suggested that the etiology of OCD might involve more widely distributed large-scale brain systems, including limbic system and the salience network [8, 9]. The salience network, composed of bilateral insula and dorsal ACC regions, has been shown to signal the external salient events such as cognitive, emotional or motivational salience so as to facilitate behavioral control in response [10]. The insular area, as part of limbic system, was indicated to functionally connect with the CSTC circuit [11, 12]. Also, previous event-related fMRI studies showed that OCD patients had altered functional activation in insular regions compared with healthy controls during cognitive and affective tasks [13–15].

Compared to event-related fMRI, the resting state fMRI measures the intrinsic functional organization of the brain, which extends the investigation into a more widely distributed brain network [16]. The low frequency (0.01–0.08 Hz) fluctuation (LFF) of blood oxygenation level dependent (BOLD) signal was shown to closely relate to spontaneous neural activity [17]. Most resting state fMRI studies of OCD focused on the aberrant functional connectivity within the orbitofronto-strital circuit, which relied on priori defined regions-of-interest (ROIs) and measured inter-regional correlation of neuronal variability [18–22]. The amplitude of LFF (ALFF) measures the total power of brain functional fluctuations within a specific frequency range, which reflects the intensity of regional spontaneous brain activity [23]. The ALFF value provided a useful biomarker to examine the whole brain intrinsic dysfunction, which has been widely applied in different neuropsychiatric disorders such as schizophrenia [24], depression [25], and tourette syndrome [26]. To our knowledge, only one ALFF study was conducted in OCD and altered spontaneous cerebral activity was observed in bilateral OFC and ACC regions [27] . However, the recruitment of recently medicated OCD patients might affect the results.

In this study, we explored the whole brain intrinsic cerebral activity using the ALFF measurement in unmedicated OCD patients and compared it with healthy controls. With interest in CSTC circuit and salience network, we tried to find the association between ALFF alteration and the clinical symptom severity as an informative biomarker for OCD pathophysiology.

## Methods

### Subjects

Twenty-eight OCD patients and twenty-eight healthy subjects participated in this study, matched for age, gender, handedness and educational status. Five patients and five healthy controls data were excluded due to excessive head motion during scanning (see image preprocessing section). The demographic and clinical information for the remaining 46 participants were illustrated in Table 1. The patients aged between 18 and 54 years (32.09 ± 10.55 years). All subjects are right-handed, and have junior high school or higher education level. 16 of the OCD patients were drug-naïve, 7 patients had a history of treatment with psychotropic medications. All patients did not take any medication that might affect the central nervous system for at least 2 months prior to MRI scans. Exclusion criteria are as follows: MRI-incompatible devices, pregnancy or lactation, severe physical illness, psychoactive substance abuse and comorbid Axis I psychiatric disorders, etc. OCD patients were recruited from the Shanghai Mental Health Center and healthy controls were recruited through local advertising. All patients were interviewed by an experienced psychiatrist with Mini-International Neuropsychiatric Interview (MINI) to provide DSM-IV diagnoses of Axis I psychiatric disorders. The MINI was utilized to determine whether they met DSM-IV criteria for OCD or other mental disorders. The whole study has been approved by Shanghai Mental Health Center Ethics Committee and each subject has signed the written informed consent before participation. All participants provided consent for the publications of anonymized individual clinical details.

**Table 1:** Demographic and clinical information on participants

Characteristics	OCD (*n* = 23)	Control (*n* = 23)	t-value^a^	*p*-value^a^
Age (years)	32.09 ± 10.55	31.39 ± 10.04	0.23	0.82
Gender	15 male, 8 female	15 male, 8 female	–	
Educational status (years)	13.87 ± 2.65	14.22 ± 2.65	−0.45	0.67
Age of onset	24.80 ± 10.68(20)	–	–	–
Duration of illness (years)	6.85 ± 4.66(19)	–	–	–
YBOCS-total	21.48 ± 5.63	–	–	–
YBOCS-obsessions	11.30 ± 2.57	–	–	–
YBOCS-compulsions	10.17 ± 3.78	–	–	–
HAMD	11.00 ± 7.91^***(22)^	1.95 ± 3.47^***(22)^	4.91	<0.001
HAMA	9.47 ± 8.07^***(19)^	0.95 ± 2.37^***(19)^	4.42	<0.001

Data are presented as mean ± S.D

YBOCS: Yale-Brown Obsessive Compulsive Scale; HAMD: Hamilton Depression Scale; HAMA: Hamilton Anxiety Scale

^a^Independent samples *t* test

^***^ *p* < 0.001, statistically significant

The OCD patients reported the symptom severity using Yale Brown Obsessive Compulsive Scale (YBOCS) [28], which scores obsession and compulsion features. For all subjects, the Hamilton Depression Scale (HAMD) and Hamilton Anxiety Scale (HAMA) were used to determine subjects’ depression and anxiety symptom level.

### MRI data acquisition

All the MRI data were acquired on a 3.0-Tesla Signa MR scanner (GE Signa Horizon LX; GE Healthcare, Milwaukee, Wisconsin) at the Ruijin Hospital in Shanghai. During MR scanning, the subjects laid supine with inflatable pillows placed between the head and coil to minimize movement artifacts. The participants were instructed to rest quietly with their eyes closed but remain awake, and avoid systematic thinking. Structural images were acquired with a T1-weighted three-dimensional (3D) inversion recovery prepared fast spoiled gradient echo (IR-FSPGR) sequence. The imaging parameters are as follows: repetition time (TR) =7.79 ms, echo time (TE) = 2.98 ms; flip angle (FA) =7°; inversion time (TI) =1100 ms; acquisition matrix = 256 × 256; slice number = 188; thickness = 1 mm; voxel size = 1 × 1 × 1 mm^3^. The functional images were acquired axially using a gradient-echo echo-planar imaging (EPI) sequence with the following parameters: TR/TE/FA = 2100 ms/30 ms/90°; Field of View (FOV) =24 cm; acquisition matrix = 64 × 64; band width = 7813Hz/pixel; slice number = 33; thickness = 4 mm; gap = 0.6 mm; voxel size =3.75 × 3.75 × 4.6 mm^3^. The obtained total volumes are 200 and total acquisition time for fMRI data is about 6.5 min.

### Image preprocessing

fMRI data preprocessing was conducted using the Data Processing Assistant for Resting-State fMRI (DPARSF) program [29], which is based on Statistical Parametric Mapping (SPM8, http://www.fil.ion.ucl.ac.uk/spm') and Resting-State fMRI Data Analysis Toolkit (REST, http://www.restfmri.net). The first 10 volumes of data were abandoned to avoid the influence of magnetic equilibrium effect and adaptation of subjects to the surroundings. Slice-timing correction and realignment for head motion correction were performed for the remaining 190 volumes. In this study, the time series of each participant’s images were realigned for head motion correction using a least squares approach and a six-parameter (rigid body) linear transformation [30], the subjects with excessive head movement (>2 mm translation or >2° rotation in any direction) were excluded. The corrected functional images were then spatially normalized to the Montreal Neurological Institute (MNI) space using the standard EPI template, and resampled to 3 mm isotropic voxels. Finally, the images were spatially smoothed with a Gaussian kernel with full width at half maximum (FWHM) equal to 4 mm.

### ALFF analysis

The calculation of ALFF was performed with REST software, following similar procedures as in previous studies [31]. Firstly, band-pass filtering (0.01–0.08Hz) was applied on fMRI data after preprocessing to reduce the very low frequency drift and high frequency noise from physiological signals such as respiratory and cardiac rhythms [17]. Then the time series was transformed to the frequency domain using fast Fourier transform (FFT) and the power spectrum was obtained. The square root of power spectrum was averaged across 0.01–0.08 Hz for each voxel, returning the corresponding ALFF. For normalization purpose, the ALFF of each voxel was divided by the global mean ALFF value [32]. In this study, the ALFF computation and further analysis were carried out within the default whole brain mask of REST.

### Statistical analysis

We analyzed the difference in clinical data between OCD patients and healthy controls using independent samples t tests by SPSS software (version 18.0, SPSS Inc., Chicago, III, USA). Moreover, to abandon the interference of head movement, the translation and rotation parameters of realignment between the two groups were compared using independent samples t tests. All the results were shown in terms of mean ± standard deviation and statistical significance was set at *p* < 0.05.

To investigate within-group whole brain ALFF patterns, voxel-wise one-sample t-tests were conducted on the individual ALFF maps for both OCD patients and healthy subjects. The thresholds for statistical significance were set at corrected *p* < 0.01 and cluster size >486 mm^3^. The multiple comparison correction was determined by Monte Carlo simulation [33] (parameters: single voxel *p* = 0.01, FWHM = 4 mm, cluster size = 486 mm^3^, and 1000 iterations) within the default whole brain mask using REST AlphaSim program [34]. Furthermore, two-sample t-tests were applied to determine the between-group ALFF difference. The thresholds were set at corrected *p* < 0.01 and cluster size >486 mm^3^ following the same correction parameters as above. To explore the relationship between ALFF measures and clinical symptom severity of OCD patients, we carried out whole brain voxel-based Pearson’s correlation analysis. The results were considered statistically significant at corrected *p* < 0.05 and cluster size >1458 mm^3^ (AlphaSim correction parameters: single voxel *p* = 0.05, FWHM = 4 mm, cluster size = 1458 mm^3^, and 1000 iterations) within the default whole brain mask.

## Results

As shown in Table 1, there were no significant differences in subjects’ age, gender, and educational status between two groups. For clinical characteristics, the mean age of patients OCD onset was 24.80 (SD = 10.68) and mean duration of illness was 6.85(SD = 4.66). The mean YBOCS total score for patients was 21.48 (SD = 5.63) corresponding to a moderate symptom severity, with mean obsession score equal to 11.30 (SD = 2.57) and mean compulsion score equal to 10.17 (SD = 3.78). In addition, OCD patients showed significantly higher HAMD and HAMA scores than healthy controls (OCD: 11.00 ± 7.91, Control: 1.95 ± 3.47 for HAMD score; OCD: 9.47 ± 8.07, Control: 0.95 ± 2.37 for HAMA score). The comparison of six translation and rotation parameters for head motion realignment showed no significant difference between OCD and control groups, as illustrated in Table 2.

**Table 2:** Head motion characteristics of OCD patients and healthy controls

Head motion parameters	OCD (*n* = 23)	Control (*n* = 23)	t-value^a^	*p*-value^a^
X translation	0.30	0.40	−1.00	0.32
Y translation	0.29	0.29	−0.05	0.96
Z translation	0.50	0.47	0.24	0.81
Pitch (degree)	0.45	0.40	0.46	0.65
Roll (degree)	0.38	0.33	0.52	0.60
Yaw (degree)	0.34	0.40	−0.61	0.55

There were no significant differences of the six translation and rotation parameters between patients and controls (all the *p* values >0.05)

^a^Independent samples *t* test

**Fig. 1:**
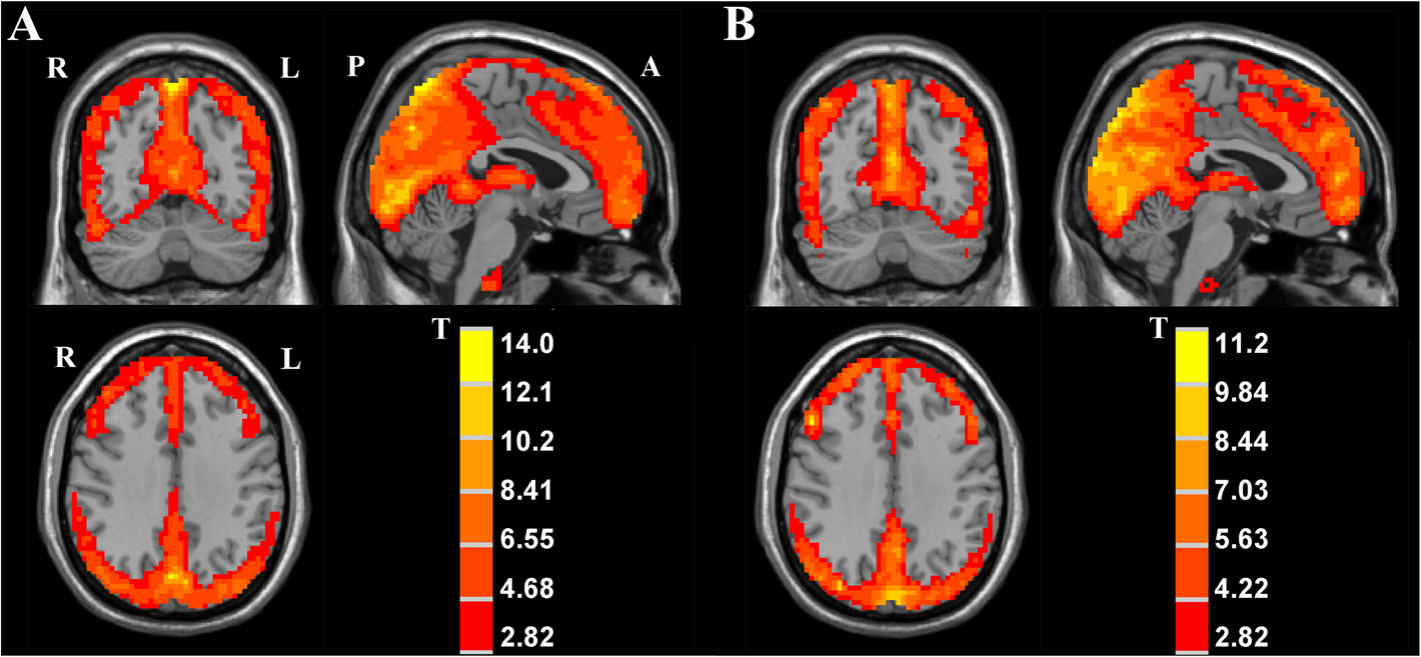
The ALFF activation patterns in OCD patients (**a**), and healthy controls (**b**) using one-sample t-test. *p* <0.01 was corrected with AlphaSim (minimum cluster size = 486 mm^3^). T-score bars indicated that the ALFF value was higher than the global mean value. R-right, L-left, P-posterior, A-anterior of the brain

**Fig. 2:**
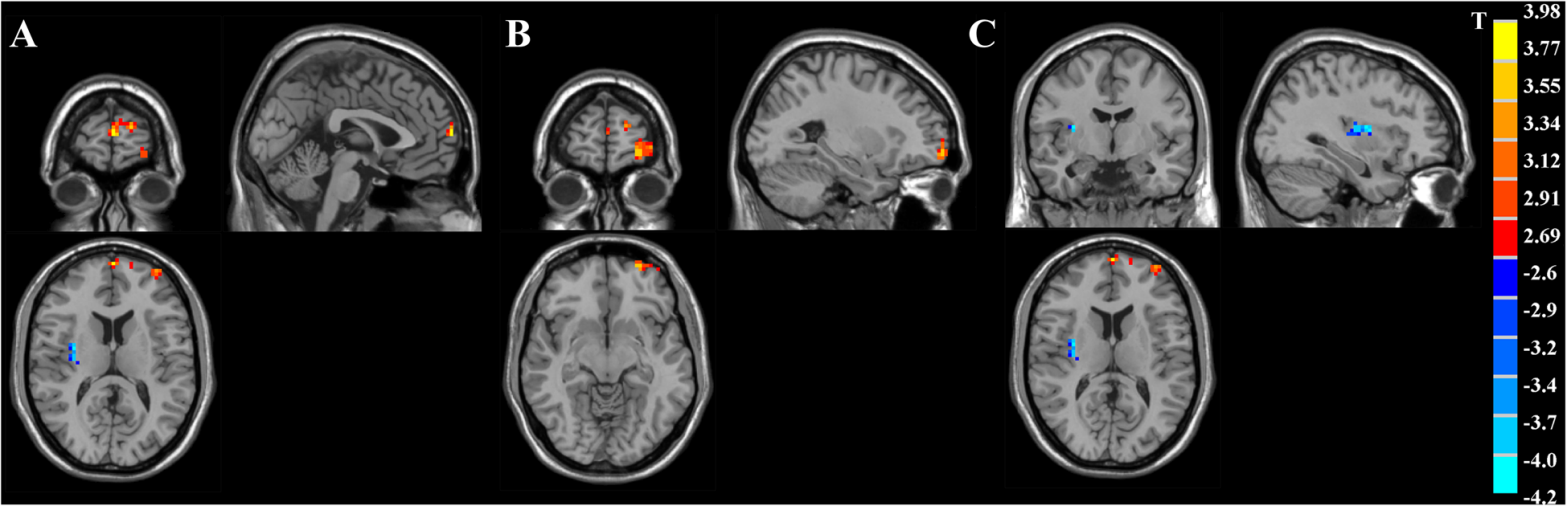
T-statistics maps showing significant ALFF difference between OCD patients and healthy controls. Patients with OCD had increased ALFF value in the left frontopolar cortex (**a**) and left OFC (**b**), with decreased ALFF in the right insula (**c**) compared with controls. p <0.01 was corrected with AlphaSim using a minimum cluster size = 486 mm^3^. T-score bar was shown on the right side of figure. Hot and cold colors indicated OCD-related ALFF increase or decrease, respectively. The left side of image corresponded to the right hemisphere

**Fig. 3:**
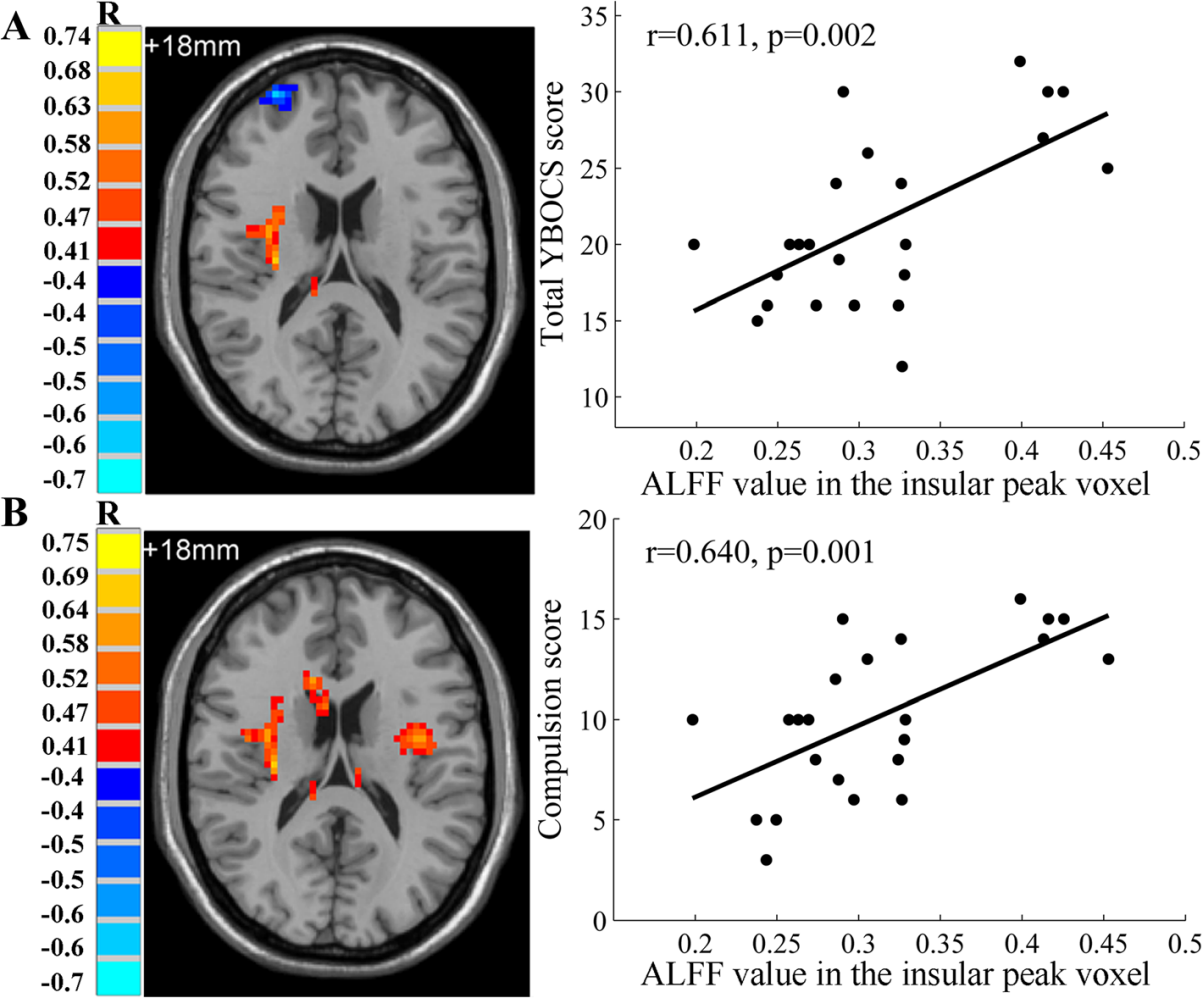
Correlation maps and scatter plots of ALFF with total YBOCS (**a**), and compulsion scores (**b**). The ALFF value of the right insular peak voxel was significantly positively correlated with total YBOCS and compulsion scores. *p* <0.05 corrected with AlphaSim using a minimum cluster size =1458 mm^3^. Axial slice displayed at z = 18 mm

The analysis of one-sample t-test displayed the ALFF activation patterns in both OCD patients and healthy controls (Fig. 1). Both groups exhibited higher ALFF value compared to the global mean value mostly within the default mode network (DMN) regions, including precuneus/posterior cingulate cortex (PCC) and medial prefrontal cortex (mPFC) areas. Moreover, the cuneus area located in the occipital lobe showed higher ALFF. When comparing ALFF difference between two groups using two-sample t-test, the patients with OCD showed significantly increased ALFF values in the left frontopolar cortex (Brodmann 10) and left OFC (Brodmann 11), compared with healthy controls. In contrast, decreased ALFF was observed in the right insula of OCD patients (Fig. 2, Table 3). We performed whole brain voxel-based correlation analysis of ALFF level and OCD symptom severity for patients. In the regions with significant group difference, the right insular ALFF value was significantly correlated with total YBOCS score (voxel volume = 2997 mm^3^; *r* = 0.611, *p* = 0.002 for peak voxel) and compulsion score (voxel volume = 1836 mm^3^; *r* = 0.640, *p* = 0.001 for peak voxel) respectively, for OCD patients. The corresponding correlation maps and scatter plots were elaborated in Fig. 3.

**Table 3:** Brain regions that showed significantly different ALFF values in OCD patients compared with healthy controls

Brain regions	Brodmann’s area	Hemisphere	MNI coordinate x y z	Peak T value	Voxels	Volume (mm^3^)
Elevated ALFF values in OCD						
Frontopolar cortex	10	Left	−1 66 13	3.67	29	783
OFC	11	Left	−27 63–9	3.51	90	2430
Decreased ALFF values in OCD						
Insula	–	Right	33–6 12	−3.83	36	972

Note that: *p* < 0.01 corrected by AlphaSim; MNI- Montreal Neurological Institute

## Discussion

The present study performed whole brain ALFF analysis using resting state fMRI to investigate intrinsic cerebral activities in unmedicated OCD adults. The OCD patients showed lower ALFF value in the right insula compared to healthy controls. Also, the whole brain voxel-based Pearson’s correlation analysis found that decreased ALFF level in the right insular peak voxel was significantly correlated with OCD symptom severity. In addition, higher ALFF values in left frontopolar and left OFC areas were observed in OCD patients. Our finding of abnormal spontaneous neuronal activity in insula advanced our understanding of OCD pathophysiology beyond the traditional CSTC circuit. The insula was shown functionally connected with dorsal ACC, which forms the salience network. To the best of our knowledge, it is the first finding about a reduced insular activity at the resting state for unmedicated OCD patients.

It is a striking finding that OCD patients showed reduced ALFF level in the right insula compared with healthy controls. As a part of salience network, insula was involved in motivation and emotion processing, which played an important role in signaling homeostatic salient events [14]. Previous neuroimaging studies have reported the insular structural and functional alterations in OCD patients. Several volumetric MRI studies found the gray matter abnormality in right insula of adult OCD patients [35–37]. In event-related fMRI studies of unmedicated OCD adults, lower right insular activation was indicated during task switching [15], while higher insular activation was reported during disgust-inducing visual stimulation [13]. In resting state fMRI studies, the insula showed decreased connectivity with striatum, which is part of the CSTC circuit [11, 12]. The excessive functional activity of CSTC circuit in OCD patients has been confirmed from a variety of neuroimaging studies [6, 7]. Our findings about reduced spontaneous functional signals in insular area suggested deteriorated consistency between insula and CSTC circuit activity in OCD. The positive correlation between right insular activity and clinical scores appears to be in conflict with our finding of reduced ALFF value in patient right insula. We presumed that the conflicts were attributed to the differences in symptom severity in OCD patients. As seen from the scatter plots in Fig. 3, the patients with high symptom severity mostly showed higher insular ALFF values, which played an important role in the positive correlation. If we subdivided the patients into two groups, i.e. moderate symptom group (YBOCS score < = 23) and severe symptom group (YBOCS score > 23) [38], we found that patients with severe symptoms showed significantly higher right insular activity than those with moderate symptoms (moderate: 0.277 ± 0.040,severe: 0.368 ± 0.066, t = -3.737, *p* = 0.003). More interestingly, we found out slightly different correlation pattern between the right insular activity and YBOCS score for each patient subgroup, e.g., for patients with moderate symptoms (YBOCS: *r* = -0.283, *p* = 0.328), and for patients with severe symptoms (YBOCS: *r* = 0.320, *p* = 0.402). Although significance was not reached, which might be due to the limited sample size, the moderate symptom subgroup showed a negative association while the severe symptom subgroup showed a positive correlation. From the analysis above, we deduced that a compensatory mechanism might be involved so that patients with severe symptoms tended to engage higher insular activity in order to reduce the difference from healthy controls. However, this finding should be interpreted with caution since our sample size is relatively small. There have been other OCD fMRI studies showing seemingly contradictory results between functional connectivity and clinical symptomatology, which was related to patients heterogeneous symptom severity [11, 39]. In order to reconcile our findings and consolidate our hypothesis, an increased patient sample size with more widely distributed symptom dimension is in need for future study.

Another interesting finding in our work is that OCD patients had increased ALFF value in the left frontopolar region (Brodmann 10), which is the most anterior part of frontal cortex. Burgess et al. implied that the frontopolar cortex was mainly involved in cognitive information processing including memory, intelligence and ability of problem-solving [40]. Recent functional imaging studies for OCD have paid increased attention to the aberrant activity in frontopolar region. In PET studies, elevated activation in bilateral frontopolar cortex during symptom provocation task was detected, and glucose metabolic rate in the left frontopolar cortex was reported to be associated with visual memory score from neuropsychological test in OCD patients [41, 42]. Increased bilateral frontopolar activation was observed in unmedicated OCD adults in event-related fMRI study and decreased activation in left frontopolar cortex was found after pharmacotherapy or behavior treatment [6, 43]. In resting state fMRI studies, unmedicated OCD adults exhibited increased functional connectivity between the left frontopolar cortex and right dorsal caudate [44]. This hyperconnectivity between the cognitive CSTC loop and limbic-associated region was proposed to suppress the maladaptive signals originating from limbic CSTC loop and our finding consistently showed a hyperactive spontaneous neuronal signal in frontopolar cortex.

As a component of CSTC circuit, the OFC is responsible for reward processing and negative effect regulation, which plays an important role in the pathophysiology of OCD [45]. Numerous neuroimaging studies have reported the OFC abnormality in OCD patients. A systematic review of voxel-based morphometry studies indicated that the structural dysfunction of OFC was highly involved in the etiology of OCD [46]. Meanwhile, hyperactive cerebral functional activity in OFC has been shown using PET and SPECT techniques [47]. From event-related fMRI studies, the patients with OCD exhibited excessive activation in the OFC area [6, 48, 49]. Recently, resting-state fMRI has been applied to assess the cerebral activity in OCD and found aberrant connectivity of OFC with ventral striatum in patients [20, 21]. In the present study, we observed increased ALFF level in OFC that is lateralized to the left hemisphere, which is in concordance with previous results for increased activation of left side OFC in OCD patients [50]. Thus, our finding of enhanced OFC spontaneous neural activity consolidated the essential role it plays in OCD etiology.

There are some limitations in this study. First, 7 out of 23 OCD patients had a history of psychotropic medications, which could have confounding effects on cerebral function. Although they were unmedicated for at least two months before participation, it is preferable if all the patients are drug naive to strengthen our findings. In addition, OCD has been demonstrated as a heterogeneous disorder with distinct neural characteristics across symptom dimensions [9]. Due to the relatively small sample size in our study, we did not investigate the influence of symptom dimensions on the neuronal activity, which could be addressed in further studies. The ALFF might be sensitive to the physiological noise in the ventricles and cisterns, which could be suppressed by an improved fractional ALFF (fALFF) approach. The fALFF utilizes the distinct frequency properties of noise and signal, and would improve the sensitivity and specificity in detecting regional spontaneous neuronal activity within the whole brain at the resting state [23]. A further study is of interest to use the fALFF approach to explore its feature in OCD pathophysiology.

## Conclusions

To summarize, we performed ALFF analysis to examine the whole brain spontaneous activity in unmedicated OCD patients using resting state fMRI technique. The OCD patients showed decreased ALFF in right insular region, and increased ALFF measures in left frontopolar and left OFC regions. Our finding of abnormal spontaneous neuronal activity in insula advanced the understanding of OCD pathophysiology beyond the traditional CSTC circuit, which might provide a new insight into the neurobiological underpinnings in OCD.

### Ethics and consent

The whole study has been approved by Shanghai Mental Health Center Ethics Committee and each subject has signed the written informed consent before participation. All participants provided consent for the publications of anonymized individual clinical details.

### Availability of data and materials

Data and materials supporting our findings in the manuscript will not be shared, due to the conflict with patients’ privacy. In addition, it was not in accordance with patients’ written informed consent.
